# COVID-19 and the importance of space in entrepreneurship research and policy

**DOI:** 10.1177/0266242620963942

**Published:** 2020-12

**Authors:** Steffen Korsgaard, Richard A Hunt, David M Townsend, Mads Bruun Ingstrup

**Affiliations:** University of Southern Denmark, Denmark; Virginia Polytechnic Institute and State University, USA; University of Southern Denmark, Denmark

**Keywords:** COVID-19, crisis, entrepreneurship, local economies, resilience, space

## Abstract

Given the COVID-19 crisis, the importance of space in the global economic system has emerged as critical in a hitherto unprecedented way. Even as large-scale, globally operating digital platform enterprises find new ways to thrive in the midst of a crisis, small and medium-sized enterprises (SMEs) nestled in local economies have proven to be fragile to shocks, causing countless local economies to unravel in the face of severe challenges to survival. Here, we discuss the role of entrepreneurship in re-building local economies that are more resilient. Specifically, we take a spatial perspective and highlight how the COVID-19 crisis has uncovered problems in the current tendency for thin contextualisation and promotion of globalisation. Based on this critique, we outline new perspectives for thinking about the relationship between entrepreneurship, resilience and local economies. Here, a particular emphasis is given to resilience building through deeply contextualised policies and research, localised flows of products and labour, and the diversification of local economies.

## Introduction

Given the impact of COVID-19, most small and medium-sized enterprises (SMEs) are encountering an array of disruptive challenges while governments struggle to enact effective policies to respond to the crisis (Cortez and Johnston, 2020; Haleem et al., 2020; Nicola et al., 2020). Evidence of the radical transformation has become ubiquitous. The importance of space has become visible in an unprecedented manner with the disruption of value chains, freeze on mobility of labour and, to a lesser extent, goods and services and even social distancing measures (Cacciapaglia et al., 2020; Kuebart and Stabler, 2020). Indeed, from a spatial perspective, the COVID-19 crisis is a brutal, unprecedented interruption of the movement of people, resources and capital. Integration and tight coupling within global value chains has suddenly become a liability in many industries, perhaps most poignantly demonstrated in the disruption of supply chains for medical supplies, the near total collapse of the tourism industry and the impossibility of maintaining in-person transactions in the service sector (Giones et al., 2020; Pantano et al., 2020). Nowhere has this become more apparent than in the market valuations of gargantuan, globally operating digital enterprises (Phillips, 2020).

For many local entrepreneurs, the long-term effects of the COVID-19 crisis will remain unknown well into the future. Importantly – and ironically – although pandemics by definition involve global spread, the effects are local as movement and mobility are restricted. In this sense, the COVID-19 crisis differs markedly from other recent crises, such as the financial crisis of 2008, which by most accounts resulted from self-inflicted, systemic economic risks that were created within the banking sector before eventually spilling over into the general economy (Harvey, 2011). In contrast, the economic devastation caused by the widespread transmission of COVID-19 originates from complex entanglements involving the biological realities of an infectious disease throughout the socio-economic structures of global capitalism (Fernandes, 2020). In a very real sense, the crisis has required humans to rethink space itself, including the socio-economic implications of space, quite literally down to the spaces occupied by individual human beings with the enactment of social distancing.

From the perspective of entrepreneurship and SMEs, two responses are of particular interest. First, on a global basis, public officials have rushed to introduce a variety of economic stabilisation measures to forestall millions of bankruptcies and small business closures (Hemmer, 2020; Palazzo, 2020; Welter et al., 2020), and jumpstarting ‘rapid recovery and growth’ (Kuckertz et al., 2020: 1). Second, local entrepreneurs and communities have come together in mutual support. We have seen communities rallying around local SMEs as a tangible sign of support for essential products, services, jobs and civic cohesion. Also, local entrepreneurs and SMEs have turned their attention to supporting at-risk groups in their communities. Central to this small business and local community fight for survival is an entrepreneurial pivoting process involving an adaptation of business models and product and service offerings to meet fundamental changes in demand (Giones et al., 2020; Hampel et al., 2020; Morgan et al., 2020) by re-building local support structures to alleviate the social costs of the crisis (see also Bacq et al., 2020; Maritz et al., 2020).

These two responses vary significantly in their objectives. The policies and support measures mostly seek to re-establish the ‘old normal’ of a globally integrated economy. The localised responses of local entrepreneurs and communities, however directly or indirectly, acknowledge how the COVID-19 crisis has exposed the vulnerabilities of the global economic system and the downsides accompanying the tight coupling of global integration. This is the basis for the creation of a ‘new normal’, one that is already being recognised by SME owners making entrepreneurial pivots to find and serve local customers in new ways (Hampel et al., 2020; Morgan et al., 2020), one that is resilient in the face of extreme uncertainty and one that incorporates a more inclusive and environmentally sustainable presence in and interaction with the economic system (Palazzo, 2020).

While the former response reproduces long-standing beliefs about the role of entrepreneurs in the development of economies, and a general neglect of the important relationship between entrepreneurship and space, the latter poses a challenge to entrepreneurship research and policy, by explicitly engaging with entrepreneurship as a localised phenomenon (Gaddefors and Anderson, 2019; Müller and Korsgaard, 2018). Rather than solely emphasising global space as the venue for entrepreneurial recovery, we point to the importance of preserving or even reconstructing local space to revitalise local entrepreneurs and SMEs since local space situates the resources and relationships that they most need to survive and thrive in an era of uncertainty (see also Castells, 1999). Thus, we highlight how local space provides a different set of opportunities for building and sustaining resilient local economies (Bristow, 2010; Christopherson et al., 2010; Hudson, 2010; Simmie and Martin, 2010; Zolli and Healy, 2012).

In this commentary, we address the challenge of rethinking the relationship between space and entrepreneurship in light of the COVID-19 crisis. In past research in the field of entrepreneurship, the importance of the spatial context in shaping the resilience of local economies has often been ignored. By implication, taking a strong spatial perspective, it is possible to reimagine new modes of entrepreneurship that are particularly conducive to recreating local economies that are more resilient and perhaps even more inclusive and environmentally sustainable.

## The importance and neglect of space in entrepreneurship research and policy

Before considering how to build resilient local economies and the role of entrepreneurship in this, it is critical to outline a few points related to how entrepreneurship research and policy have treated the intersection of space and entrepreneurship. A comprehensive review is beyond the scope of this comment, so in the following, we point to two critical tendencies that are particularly relevant in light of the COVID-19 crisis and the pressing need to facilitate the long-term recovery of local economies. Specifically, we highlight the tendencies in entrepreneurship research and policy towards thin contextualisation and towards promoting globalisation and integration in global flows.

### Thin contextualisation

As pointed out in several contributions in entrepreneurship, there has been a long-standing tendency to disregard or underplay the importance of contexts, including spatial context (Trettin and Welter, 2011; Welter and Baker, 2020). This critique is less about a disregard for, or lack of focus on spatial issues but more about how the issue has been studied. We suggest that the majority of spatially sensitive research in entrepreneurship can best be described as ‘thin contextualisation’ (see Welter et al., 2019, for a similar point; Steyaert and Katz, 2004). Detailed analyses of places such as Silicon Valley are a prominent example of this stream of research (Saxenian, 1990). Silicon Valley has simply been adopted as a role model in thinly contextualised entrepreneurship research and policy; seeking to create pulsating entrepreneurial ecosystems by essentially trying to copy–paste the structural blueprint of Silicon Valley has become the default policy option, making sightseeing tours to Palo Alto a ‘must-do’ (Blank, 2011). Myopia in entrepreneurship research and policy has followed, closing spaces for alternative policies or relegated them to second-tier policy areas (Mayer, 2011).

Within this logic, urbanised high-tech, high-growth and high-risk models of entrepreneurship have been promoted as standard despite the fact that very few places have the infrastructure, resource base and investment opportunities needed to come anywhere near the kind of entrepreneurial ecosystem we see in hubs such as Silicon Valley. In fact, like much else in entrepreneurship, most places are really in what may be referred to as the long tail of innovation and economic development and face a number of limiting factors such as the dominance of SMEs, the presence of precious few large but often externally owned enterprises, a dependence on incremental and process innovation, few educational and support organisations as well as weak and mostly local knowledge networks (Isaksen, 2015; Tödtling and Trippl, 2005). The notion of second-tier or organisationally thin regions (Mayer, 2011; Tödtling and Trippl, 2005) highlights the fragility of these contexts and why, despite the best intentions of policy makers and local leaders, development plans building on the Silicon Valley template fail to replicate the success of the few entrepreneurial hotbeds.

The myopia of thin contextualisation, as exemplified in the Silicon Valley template, ignores othered spatial manifestations such as rural entrepreneurship (Gaddefors et al., 2020; Hunt et al., 2019). Notably, an important implication of this is the application of evaluative measures involving growth and job creation, which are considered generic and aspatial. Yet, such measures are decidedly more meaningful in dynamic urban centres where agglomeration effects, competition and resource availability create the foundation for such impact. In rural communities, growth ambitions are frequently – and reasonably – secondary to maintaining communities and creating social and cultural value in the local areas (Alsos et al., 2014; Bosworth, 2012; Korsgaard et al., 2015b), and the high-tech and high-growth policy template is out of touch with the realities of de-population, de-skilling and centralisation experienced in many peripheral areas.

### Promoting globalisation and integration in global flows

Closely aligned with the above-mentioned tendency for thin contextualisation, much of the research and policy conversations in the field of entrepreneurship have focused on the importance of global economic integration for enhancing the economic vitality of local and regional economies. Under the banner of Porterian specialisation, the logic of these approaches centres on local economies and, ostensibly, local businesses to identify their comparative advantages as the means for enhancing their standing in the global economic system (Porter, 1996). This classic framework focusing on clusters of economic activity and the comparative advantage of regions has emphasised three related imperatives: (1) integrating local economies into global markets, (2) competing with other regions on the basis of local specialisation and (3) attracting outside investments and enterprises through, for example, low taxes and economic incentives.

In its ideal state, the competitiveness paradigm will lead to a set of global optima where local resource bases are combined with outside capital, enterprises and skilled workers as seen in specialised clustering around information technologies, aerospace, biotech and other industries in places such as Silicon Valley, Boston and Cambridge (Porter, 1998). Entrepreneurial ventures are essential drivers in this process. Of particular value here are new ventures that extend the specialisation of the local economy, generating further spillover effects directly into the focal industry or indirectly in support of it.

To further this agenda, many countries have adopted a neoliberal agenda of deregulation, internationalisation and global integration in order to facilitate the entry, engagement and integration of local businesses into the global economy. Typically, these policies direct investments primarily into current industries of strength; the evaluative measure is high growth in economic profits and new jobs, preferably high-skilled jobs. The race to the bottom of corporate taxes and the bidding wars between locales in attracting corporate headquarters stand as testament to the extreme manifestations of this paradigm (Mazzucato, 2015; Streitfeld, 2020; Thoma, 2020). For example, witness the dizzying competition by hundreds of locales to land Amazon’s HQ2 by offering billions in tax breaks. Ironically, in the case of Amazon, the final decision ultimately hinged on a completely different set of decision factors, stemming mostly from the robustness and vitality of the local economic ecosystem in Arlington County, Virginia, fuelled in part by a robust university system and a strong local infrastructure.

Despite the fact that entrepreneurs are spatially positioned and the agglomerated outcome of entrepreneurship has spatial consequences, the spatial dimension in this view is somewhat implicit (Stam and Lambooy, 2012) or obscured (Andersson, 2005), as the integration of local enterprises into the global economy is meant to dissolve the spatial barriers in order to secure the free flow of information, resources, capital and production, with urbanisation and agglomeration following naturally when economically conducive (Gordon and McCann, 2005). The pro-growth advantages of a combination of globalisation and entrepreneurship have been touted loudly and insistently; it needs no repetition. However, the COVID-19 crisis has laid bare the vulnerabilities of global integration leaving many local economies devastated as they relied heavily on the influx of investments, supplies, tourists and so on, as well as global output markets (Kuckertz et al., 2020). This fragility has been re-enforced and worsened by the economic policies of competitiveness over the past decade (Bristow, 2010; Christopherson et al., 2010; Hudson, 2010; Simmie and Martin, 2010).

## (Re)discovering and (re)building resilient local economies

As the foregoing discussion suggests, the COVID-19 crisis has exposed a variety of problems in the global integration agenda of eliminating local space as the means to ensure integration of enterprises into global markets. The starting point for constructive reassessment involves the fundamental shift from an exclusive focus on global competitiveness towards an emphasis on the importance of the local spatial context in shaping the resilience of local economies (Frazier et al., 2013; Hudson, 2010; Modica and Reggiani, 2015). As noted earlier, global integration is built on comparative advantages possessed by local communities which often yields low prices and the efficient concentration of productive resources and outputs wherever efficiencies can best be achieved. However, these global optima constitute an idealised state. The reality on the ground is altogether different, as hyper-specialisation in local environments, especially in the COVID-19 era, leaves local environments in a bind if global systems freeze, falter and fail (Griffith-Jones and Ocampo, 2009; Ravetz, 2005). As such, the social fissures, economic inequities, steep hidden costs and other underlying dangers attendant to the adoption of a competitiveness approach have been laid bare through the crisis. In particular, locales now bear witness to the manner in which highly concentrated, long-lived investments in one or few specialisations and industries may inadvertently sacrifice economic sustainability in support of global value and supply chains. While 2008 saw supply-side shocks and deleterious impacts wreaked upon finance and manufacturing, COVID-19 impacts have involved both supply-side and demand-side forces that have decimated the service sector, which accounts for more than 80% of Western economies (Fernandes, 2020). In retrospect, the competitive positioning focus on comparative advantage and global optima created fragility to external shocks, emphasising the trade-offs involved in building local economies (Hudson, 2005, 2010; Hudson and Maioli, 2010; Korsgaard et al., 2016).

The notion of resilience is critical as global optima are, by definition, linked to and reliant upon systems-level thinking in which locales are subordinate contributors to and beneficiaries of a global system of efficiency. As noted from the outset, the logics supporting this approach have only been strengthened by economic responses to the crisis, wherein globally operating e-commerce enterprises are notably better equipped to provide immediate relief to isolated consumers and fractured supply chains. Thus, while the long-term solution may reside in elevating local self-reliance, systems predicated on global metrics of comparative advantage are largely ineffectual to local impacts. This creates socio-economic fragility at the local level through the inherent dependencies of consumers and SMEs being reliant upon a global system that emphasises fragmented production and a high degree of balkanised specialists. While this system excels at generating cost-saving efficiencies, it concomitantly accelerates the loss of local economic resilience (Zolli and Healy, 2012).

Looking to the future, the recent shocks provide cautionary insights, though it is unclear whether they will elicit meaningful change in attitudes and policies related to the comparative advantage and global optima paradigm. Although the rush to embrace remote, e-commerce solutions is clearly anathema to local economic resilience, there are also dangers in rushing to fuel short-term growth and peak efficiency, post-crisis, through a ‘copy–paste approach’ to competitiveness that is likely to simply repeat the neglect of space that caused the loss of resilience in the first place. (Re)building resilient local economies that are less susceptible to the adverse effects of external shocks and endogenous crises involves a long-term effort to shift the collective mind-set to one that is receptive to a sacrifice in short-term growth and peak efficiency aims, in order to strengthen fragile locales to better withstand the threat of catastrophic impacts (Zolli and Healy, 2012). If this dire state of affairs dominated the past and present, is there any prospect for a decidedly different future? In the following section, we outline a tentative new role for entrepreneurship in the effort to (re)build local economies that are more resilient. Properly conceived, implemented and supported, this may serve as a guide for policy making, education and training of future entrepreneurs and for directing public and private investments in entrepreneurship that builds local resilience.

## A new role for entrepreneurship

The new role for entrepreneurs in (re)building resilient local economies raises important questions for how new enterprises might leverage and build on their ties to the local spatial context as the means for improving their resilience. ‘Remember what small businesses do’, said Mohamed El-Erian, former CEO of investment behemoth, PIMCO, in response to the pandemic’s impact on SMEs, ‘They’re not just important employers, they also are the main way to have inclusive capitalism, an inclusive market-based system’ (Stankiewicz, 2020). While the foundation of this inclusiveness has traditionally emphasised the integration of SMEs into globalisation efforts, the global financial crisis of 2008 and COVID-19 crisis emphasise the critical need to rethink the sources of sustainable local resilience.

Entrepreneurs and entrepreneurship scholars can play a central role in shifting policies and priorities through three interrelated elements. First, both the scholarship and practice of entrepreneurship can further develop a more deeply contextualised conception of ‘place’, reflecting greater attentiveness to the idiosyncratic characteristics of locales, rather than benchmarking to a generic model that propounds comparative advantage with a view towards implementing thinly contextualised templates and favouring global optima. Second, scholars, practitioners and policy makers can emphasise the creation and integration of local resources and the localised flow of capital, products and labour as a significant supplement to globalisation policies. Third, meaningful improvements in local resilience require investments in the diversification of local economies, with a particular emphasis on creating and stabilising community-oriented, meso-level organisational forms to promote local integration and resilience.

### Deep contextualisation

Deep contextualisation (see Hunt et al., 2019, for a similar point emphasising radical contextualisation) involves the identification and integration of five factors that are essential to understanding the manner in which a locale constitutes an entrepreneurial context: spatial, temporal, social, institutional and commercial (Welter, 2011; Zahra and Wright, 2011). As such, each locale is a socio-material configuration of idiosyncratic constraints and enablers, whose combinations and interactions influence the types and extent of entrepreneurship, as well as the value and impact of various entrepreneurial activities on the sustenance and development of local economies, places and communities (Hudson, 2001; Korsgaard et al., 2015b). As noted above, scholarly approaches employing thin contextualisation have focused on the hotbeds of high-growth, high-tech entrepreneurship, with the aim of generalising the antecedents and outcomes of these agglomeration spaces into contemporary theories of entrepreneurship. This emphasis on high-growth, technology-based ventures has indelibly influenced scholarly conceptions of the role played of entrepreneurship in economic development (Malecki, 1991) as well as the bias towards investing in technological prowess through policy actions (Scherer and Harhoff, 2000). In such cases, the contextualisation is ‘thin’ because it views locales as deriving their meaning and purpose from the manner in which they fit into the achievement of global optima. Unsurprisingly, thin contextualisation results in the development and promulgation of economic development templates that are principally adopted with the aim of aligning national or regional systems of entrepreneurship or entrepreneurial contexts towards the attempt to replicate the ‘hotbed’ model (Ács et al., 2014; Brown and Mason, 2017; Muñoz and Kimmitt, 2019).

The concept of deep contextualisation acknowledges two important realities: homogenisation is impossible to achieve and undesirable even if it could be. Local spaces, places, communities and economies are influenced – albeit in an evolving fashion – by distinctive contextual factors that have very long tails, rendering ineffectual the thin conceptualisation of entrepreneurship that has dominated research and policy over the past decades. Instead of converting general theories of entrepreneurship, and its role in economic development, into homogenised policies aimed at supporting ‘gazelles and unicorns’ (Aldrich and Ruef, 2018) through an emphasis on high-performing entrepreneurial ecosystems within wildly heterogeneous contexts, deep contextualisation encourages policies and imagery of entrepreneurship derived from the history, spatial conditions, resource endowments and needs of local economies.

Bottom-up programmes such as the EU LEADER initiative are well suited for this reorientation in that they allow for localised analysis of the needs and potentialities of local places and communities. Therefore, they can involve multiple forms of stakeholders so that such programmes are more likely to be conducive to building resilient local economies. Two important conditions for this, however, need to be highlighted. First, it is important that local development strategies are not unduly influenced by myopic conceptions of what constitutes successful economic development, that is, high-tech, cluster-based formations that may or may not translate well to local culture, geography and other conditions. Even within the context of urban areas, resilience is best achieved by considering the benefits of idiosyncrasies and achievable appropriateness, rather than ill-fated attempts to emulate Silicon Valley. Local resilience is a function of what makes places and communities different from each other, rather than force-fitting homogenised sameness, which leads to fragile local economies and a loss of resilience. Second, it is vitally important to achieve agreement on the need to adjust the outcome measures of investments in entrepreneurship and SME development. Measuring job creation and enterprise growth makes for too simple comparisons and easy political justification; yet, these measures vary in relevance and usefulness.

Regarding both of these conditions, there is evidence that scholars have recognised the need to shift away from a sole preoccupation with ‘gazelles’ and ‘unicorns’ as cornerstone of economic development (Aldrich and Ruef, 2018). For example, emerging research on ‘makerspaces’ (Browder et al., 2019) suggests a growing awareness that entrepreneurship as a field and as an endeavour is barren without contextualisation of entrepreneurial ideation and action that accounts for how and why entrepreneurs engage in social exchange, knowledge creation and novel production, involving products formally the exclusive provenance of large enterprises.

All communities need jobs and growth, but these are neither the only needs nor the only drivers of resilience. Rather, locally relevant aims involve the kinds of decisions and trade-offs that lead to a renewed emphasis on greater self-sufficiency and a more diverse, sustainable, homegrown economic base. For example, in rural areas, the decision to support a local grocery may be much more important to a community if the store represents the last remaining local alternative, compared to urban areas that are saturated with supermarkets, and where new entrants will quickly emerge if an existing supermarket closes (Bailey, 2010; Morton and Blanchard, 2007). Conversely, in many urban areas, job creation may be less of a persistent concern, with affordable housing, or equal access to healthcare services being greater concerns. As both scholars and social commentators have emphasised regarding the pandemic, exogenous shocks and endogenous crises certainly cause damage, but, much more so, they reveal damage that already exists in spaces that have lost resilience (Bacq et al., 2020; Kuckertz et al., 2020). Whatever the local susceptibilities, from rural to urban, the (re)building of resilient local economies requires diversified, place-specific approaches to policy development that contemplate the essence of deep contextualisation. Notably, local policies cannot stand alone. International efforts to level the playing field by limiting the advantages of global enterprises are essential. Tax evasion by large enterprises and the failure of the global system in internalising the environmental and social externalities of globalised production and consumption are important examples of how local enterprises face an uneven playing field and uphill battle when competing (Morgan, 2016; Müller, 2019; West, 2018). Addressing these problems is imperative.

### Local resources and localised flows of capital, products and labour

As noted from the outset, the COVID-19 crisis has demonstrated vulnerabilities in the global flows of commodities, goods and services, and in particular how interruptions of those flows have a pronouncedly adverse impact on local economies. The effects are driven by catastrophic collapses in both supply and demand. For instance, locales that are heavily reliant on global factor markets – as with enterprises that are dependent upon suppliers from China to deliver components for production – saw many of those enterprises pushed to extinction due to supply-side dependencies. Meanwhile, locales tightly linked to globally derived output markets – as with those supporting seasonal, tourism-based ventures – are struggling due to a severe, sudden and potentially prolonged drop in demand. This is the quintessence of socio-economic fragility arising from over-specialisation, driven by a comparative advantage approach that forestalls the emergence of diverse entrepreneurial activity (Kellman and Shachmurove, 2011).

While globally integrated local economies can, ceteris paribus, achieve higher outputs than local economies with less integration, the vulnerability, arising through a lack of diversity and exposed by successive crises, makes a strong case for the usefulness of local resources for local markets and the intrinsic benefits of self-reliance in mitigating the exposure that comes with global integration (Hudson, 2010). The use of local resources to compensate for difficulties in accessing global markets is more prevalent among rural entrepreneurs, where sustainable, context-relevant business venturing is the norm. Scholars have observed that such an approach, especially as it has been manifested in European countries, demonstrates the efficacy of aims to (re)build more resilient local economies (Kitchen and Marsden, 2009), even in urban metropoles.

The transition to local flows is far from costless. Investments as well as a fundamental change in mind-set are required for policy actions to find fertile ground. Without question, local production of food, vital goods and services, decentralised systems of energy production, digital infrastructure, health and education services and other efforts to develop and deploy local resources for local use come at a higher short-term cost (Zolli and Healy, 2012). However, the tension between global optima and local resilience is not an all-or-nothing, binary proposition. In the end, some greater degree of local economic diversification provides a material hedge against global disruptions (Hudson, 2010; Korsgaard et al., 2016). The support of entrepreneurs who extend the use of local resources by building diversified local markets, predicated on local production and use, may well be a better way forward than committing solely to a default valorisation of born globals with a patently high-tech orientation.

### Diversification of local economies

Extending the point made above, another critical determinant of the ability to (re)build local economies involves the need to balance specialisation with diversification in local economies. The Porterian logic of global integration calls for specialisation exploiting local strengths. This logic has merit as part of a composite, policy programme and has been shown to be highly instrumental to the development of high-growth economies (Kellman and Shachmurove, 2011). However, while there is a short-term cost to (re)building more resilient local communities, there is a long-term price tag that accompanies ill-fated, thinly contextualised over-investments in seeking to force-fit high-velocity, high-tech foci for the sake of comparative advantage aims. Moreover, this homogenised blueprint for economic development suffocates the entrepreneurial ecosystem it is intended to jump-start. Existing research has documented the weak track record of government-sponsored, new venture incubators, wherein key contextual factors are ignored, while locales attempt to replicate unachievable high-tech agglomeration models (Tamasy, 2007) – a folly that has repeatedly resulted in empty, ‘white elephant’ structures and a mountain of long-term indebtedness (Isenberg, 2010). Instead, a focus on diversifying the use of local resources and strengths, expanding into new industries and building local markets provides resilience that may not forestall the next inevitable crisis, but it will blunt the extent of its adverse impacts.

Here, too, there are compelling examples of alternative pathways that can be drawn from rural contexts, where the idiosyncrasies of ‘place’ are more long-lasting and highly prized. While it would be wrong to valorise rural over urban entrepreneurial logics amid the many hardships and entrenched challenges of rural locales, there are long-overlooked approaches to space and place in rurality that offer intriguing pathways to sustainable local resilience (Hunt et al., 2019; Korsgaard et al., 2015a). With an eye towards the intrinsic benefits of diversity, rural entrepreneurs often build and nurture portfolios of ventures using the resources of locally embedded family farms and the local area. This, in turn, creates jobs for the community, but the approach also creates meaning for family members. Such practices make families and communities less exclusively reliant on the main farming activity, while providing new avenues for income as a hedge against cyclical pressure on the core farming business (Alsos et al., 2014; Carter, 1998, 2001). Processes of bricolage and exaptation are central to these well-documented processes (Gaddefors et al., 2020; Garud et al., 2018); yet, unlike exaptation in the technological domain (Andriani and Cattani, 2016; Ching, 2016; Dew and Sarasvathy, 2016; Garud et al., 2016), diversification in the rural context provides the means to establish resilience in the local economy wherein the value is determined as much by system-level resilience as by profitability or the resource-use efficiency of entrepreneurial enterprises. While rural contexts are uniquely informative on these points, there are similar contextual anecdotes emanating from urban areas that provide similarly compelling insights (see Hunt et al., 2019), though they are often more difficult to translate to resource-constrained and infrastructure-poor locales. The common denominator is not the population intensity, but the recognition that resiliency and self-sufficiency are notable aims for socio-economic well-being.

## Conclusion

This commentary outlines a spatial perspective on the role of entrepreneurship in the development of local economies in light of the current COVID-19 crisis. We argue that the crisis has brought to light the importance of space, and how the treatment of space has suffered from undue superficiality in existing research and policies related to entrepreneurship. Instead of replicating past errors of omission, we encourage re-framing the crisis as an occasion to reconsider the role of entrepreneurship as an essential ingredient in the effort to build resilient local economies. As we have emphasised, this is as much a matter of mind-set as economic wherewithal. Short-term, concerted efforts to build more resilient spaces will cause locales to become less competitive, as a consequence of the indomitable benefits of specialisation and comparative advantage. However, as the crisis has made clear, unless one owns or operates a globally scaled e-commerce enterprise, the long-term price tag for local fragility is a catastrophic extinction of SMEs and a locale’s entrepreneurial context.

The shift to local resilience involves willingly eschewing tight integration with global markets to looser forms of coupling. The ‘new normal’ for more self-sufficient communities necessarily includes deeply contextualised, highly heterogeneous policy programmes, support of local markets and flows, and diversification of the local resource use. Unlike other responses that are hesitant towards globalisation – the notion of trade tariffs as an example – we have asserted that efforts to globalise must be balanced with efforts to support forms of entrepreneurship that engage the local economy and community as richly diverse platforms for novel venturing. In searching for a future that is more promising than the past, we must look to places other than just Silicon Valley for inspiration and identify other ideal types than the high-tech and high-growth gazelles. We have seen in this crisis the starkness of survival-based isolation and the dependence we have upon global supply chains that are ineffectual to local differences. As the isolation has persisted, individuals and communities have become increasingly beholden to the Apples, Googles, Amazons and Facebooks of the global supply networks – enterprises that may satiate short-term needs, but which circumvent the vitality of spaces that will sustain us when COVID-19 has finally been tamed. When at last, community members tire of Door Dash and a mailbox-centric, Amazon-provisioned existence, they will amble outside to patronise local restaurants, stores, hotels and farms, and may be shocked to find that many local SMEs no longer exist. It will be the important work of policy-enabled and inspired entrepreneurs to fashion new local economies that aspire to greater self-sufficiency and resilience, and correspondingly less reliance on a highly specialised global supply chain for which the isolating effects of pandemics are less of a crisis than they are a growth opportunity. Instead, a focus on building sustainable locales through a vibrant context for SMEs is a stronger foundation upon which to build and thrive.
